# Deep Learning-Based Natural Language Processing for Screening Psychiatric Patients

**DOI:** 10.3389/fpsyt.2020.533949

**Published:** 2021-01-15

**Authors:** Hong-Jie Dai, Chu-Hsien Su, You-Qian Lee, You-Chen Zhang, Chen-Kai Wang, Chian-Jue Kuo, Chi-Shin Wu

**Affiliations:** ^1^Intelligent System Laboratory, Department of Electrical Engineering, College of Electrical Engineering and Computer Science, National Kaohsiung University of Science and Technology, Kaohsiung, Taiwan; ^2^School of Post-Baccalaureate Medicine, Kaohsiung Medical University, Kaohsiung, Taiwan; ^3^National Institute of Cancer Research, National Health Research Institutes, Tainan, Taiwan; ^4^Department of Psychiatry, National Taiwan University Hospital, Taipei, Taiwan; ^5^Big Data Laboratory, Chunghwa Telecom Laboratories, Taoyuan, Taiwan; ^6^Taipei City Psychiatric Center, Taipei City Hospital, Taipei, Taiwan; ^7^Department of Psychiatry, School of Medicine, College of Medicine, Taipei Medical University, Taipei, Taiwan

**Keywords:** deep learning, natural language processing, text classification, patient screening, psychiatric diagnoses

## Abstract

The introduction of pre-trained language models in natural language processing (NLP) based on deep learning and the availability of electronic health records (EHRs) presents a great opportunity to transfer the “knowledge” learned from data in the general domain to enable the analysis of unstructured textual data in clinical domains. This study explored the feasibility of applying NLP to a small EHR dataset to investigate the power of transfer learning to facilitate the process of patient screening in psychiatry. A total of 500 patients were randomly selected from a medical center database. Three annotators with clinical experience reviewed the notes to make diagnoses for major/minor depression, bipolar disorder, schizophrenia, and dementia to form a small and highly imbalanced corpus. Several state-of-the-art NLP methods based on deep learning along with pre-trained models based on shallow or deep transfer learning were adapted to develop models to classify the aforementioned diseases. We hypothesized that the models that rely on transferred knowledge would be expected to outperform the models learned from scratch. The experimental results demonstrated that the models with the pre-trained techniques outperformed the models without transferred knowledge by micro-avg. and macro-avg. F-scores of 0.11 and 0.28, respectively. Our results also suggested that the use of the feature dependency strategy to build multi-labeling models instead of problem transformation is superior considering its higher performance and simplicity in the training process.

## Introduction

Currently, the diagnosis and classification of mental disorders are commonly based on the International Statistical Classification of Diseases and Related Health Problems (ICD) and the Diagnostic and Statistical Manual of Mental Disorders (DSM) system. These diagnostic criteria are derived from the clinical observation of the symptoms, signs, and course of mental diseases. The differential diagnosis of mental disorders is quite important because the decision for treatment selection and prediction of prognosis are dependent on the accuracy of diagnosis. However, making a diagnosis is not easy because symptoms are commonly shared among these diagnoses. For example, depressive symptoms are often observed in patients with bipolar disorders. If a psychiatrist were to ignore previous hypomanic or manic episodes, bipolar disorders would be misdiagnosed as depressive disorders. The use of antidepressants for bipolar depression might also induce mood swings or a manic episode. Another example is that depressive symptoms are frequently observed in patients with dementia. The differential diagnosis between pseudo-dementia (as a result of cognitive impairment due to depression) and dementia might be quite difficult. Antidepressant treatment might increase the risk of cerebrovascular accidents among these patients (1).

On the other hand, the natural language processing (NLP) community is now witnessing a dramatic paradigm shift toward pre-trained deep learning-based language representation models such as bidirectional encoder representations from transformers (BERT) (2), which achieve state-of-the-art performance for tasks such as question answering, sentiment classification, and similarity modeling. For example, Peng et al. (3) demonstrated that the BERT model pre-trained on PubMed abstracts and MIMIC-III clinical notes outperformed state-of-the-art models on ten biomedical benchmarking datasets. Recent advances in NLP based on deep learning motivated us to ponder whether it could be applied to develop a classification model that could facilitate psychiatrists' diagnoses and improve the accuracy rate of diagnoses even with a small dataset. We approached the research question by using a dataset containing 500 discharge summaries to learn the psychiatrists' diagnoses of five common mental disorders, including major and minor depression, schizophrenia, bipolar disorder, and dementia based on unstructured electronic health records (EHRs). We hypothesized that the models with transferred knowledge are likely to outperform the models learned from scratch. In clinical applications, these NLP models could assist with diagnosis and improve the accuracy, especially in the case of primary physicians who are not familiar with psychiatric diagnosis. Furthermore, it might be possible to use NLP models to detect psychiatric illness early in social media.

In this study, we considered the aforementioned task as a text classification task with the goal of assigning five labels to a given discharge summary in an EHR. Four advanced NLP network architectures based on deep learning along with a variety of pre-trained models were adapted to study the feasibility of applying them to classify the above-mentioned five diagnoses.

## Methods

### Corpus and Annotations

With the approval of the Research Ethics Committee of the National Taiwan University Hospital (NTUH-201610072RINA), the corpus compiled in our previous work (4) was used in this study. This corpus contains 500 unstructured discharge summaries sampled from the psychiatric unit of the Integrated Medical Database of National Taiwan University Hospital (NTUHIMD) and given a principal psychiatric diagnosis (ICD-9-CM codes 290-319 or ICD-10-CM codes F00-F99). All personal information of the patients included in the corpus was de-identified.

Each discharge summary is presented in the form of unstructured texts, which may include the demographic descriptions, present illness, physical and mental examinations, progress notes, laboratory and image examinations, prescription records, and medical procedures for one patient. The summaries were manually chart reviewed by board-certified clinical psychiatrists (CJK and CSW) to determine whether the patients had schizophrenia (SCZ), bipolar disorder (BPD), major depressive disorders (MDD), minor or other depressive disorder (mDD), and/or dementia (DD). After chart review, each summary in the compiled corpus was categorized as being one of the aforementioned diagnoses.

**Figure 1:**
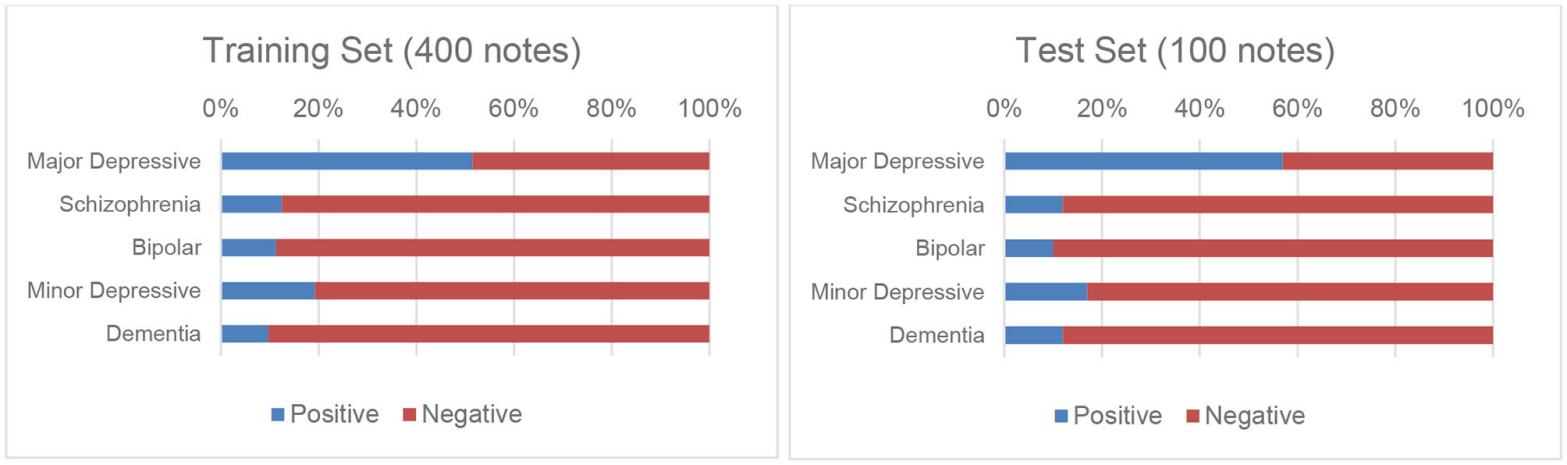
Distributions of the five disorders in the training and test sets.

Although the accuracy of such a diagnosis was not as valid as that made on the basis of a structured diagnostic interview, the agreement between psychiatrists was acceptable. The values of Cohen's kappa ranged from 0.73 to 0.91. Generally, the agreements between the diagnosis of major depressive disorder (0.82), schizophrenia (0.90), and dementia (0.91) was almost perfect, and that of bipolar disorder and minor depression was acceptable (0.73 and 0.74, respectively). The detailed annotation process and kappa values can be found in our previous work (4). We followed the same procedure we previously employed (4) to randomly divide the corpus into training and test sets, each of which contained 400 and 100 notes, respectively. Figure 1 presents these distributions graphically.

### Pre-processing

The EHRs include several sections that are irrelevant to this work, such as laboratory and image examinations, prescription records, and medical procedures. However, the symptoms and clues for diagnosing the five disorders usually appeared in the “Brief History” (BH) and the “Physical and Mental Status Examination” (PME) sections. Because the unstructured texts in each summary were organized into several pre-defined sections in NTUHIMD, we were able to develop a pre-processing procedure to extract only the texts from the BH and PME sections to train and test our models. This procedure was designed to prevent the learned models from occasionally considering noisy information from irrelevant sections to make their predictions. Note that not all summaries in our corpus contained both the BH and PME sections; in particular, 99.5 and 98.5% of the notes contained the BH and PME sections, respectively.

The summaries were then processed by our clinical NLP toolkit (5, 6) to segment them into sentences and their corresponding tokens in lowercase. Although most of the sentences were represented in English, sentences containing a mixture of English and Chinese descriptions were also included. The Chinese characters were removed during pre-processing to simplify subsequent text analysis. Subsection titles such as “Family History” and “History of Substance Use” were recognized by using regular expressions and were also removed to reduce the length of the text. Finally, text normalization was applied by mapping selected subsets of words and phrases into their representatives. For example, the words “w/o,” “w/” were normalized to “without” and “with,” respectively.

### Deep Learning Models for Screening Psychiatric Patients Based on Textual Data

**Figure 2:**
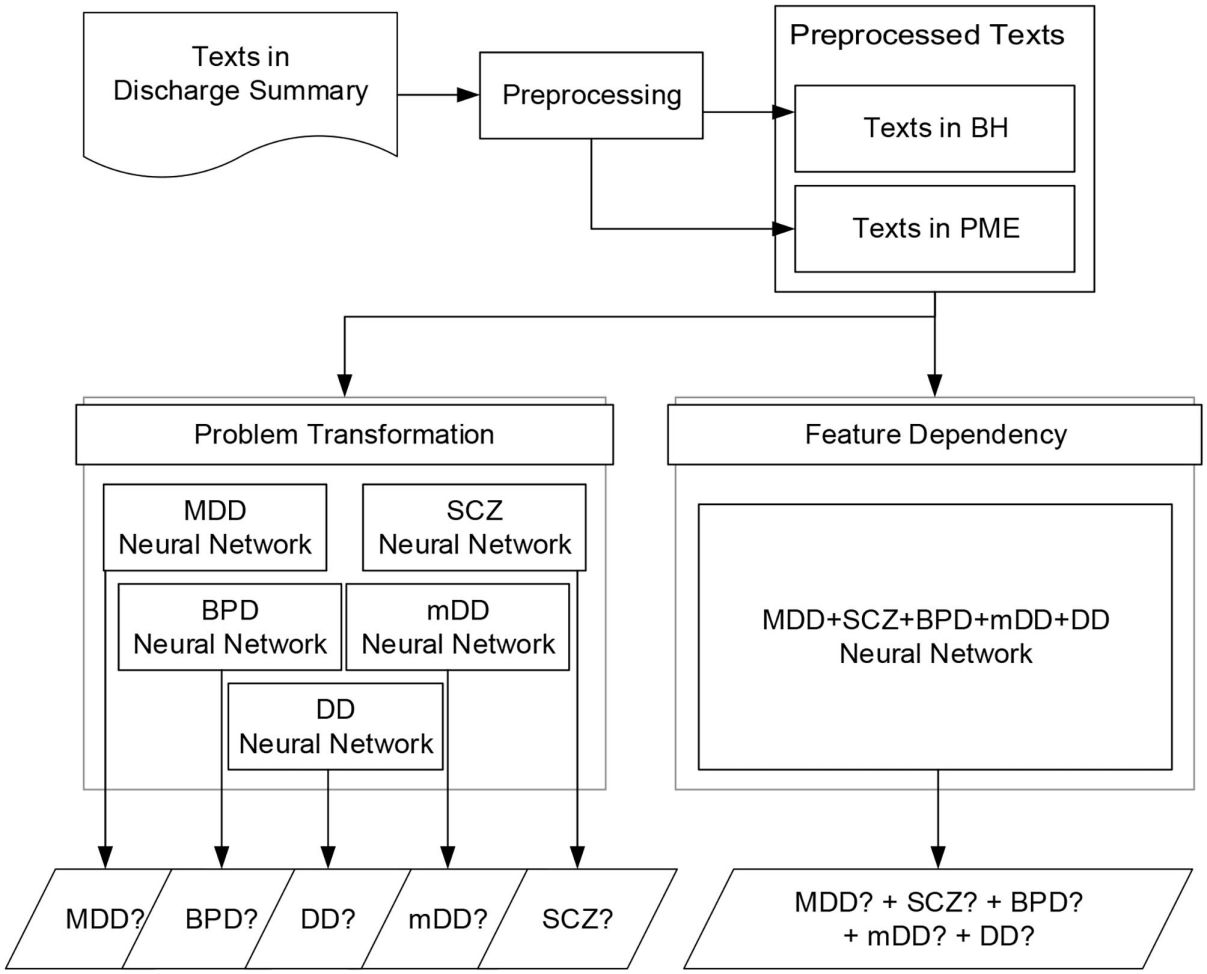
Flowchart of the screening process of the neural networks based on the two methods: problem transformation and feature dependency.

In this section, we first provide an overview of the developed deep learning models.^1^ We then elaborated on the architectures of the developed models in the following subsections. Figure 2 summarizes the flowchart for the screening process based on the developed models. The inputs of our models are the pre-processed texts extracted from the discharge summaries. The outputs are the probability distributions of the five disorders. A positive diagnosis is determined if the outcome of the output layer of the network is larger than a specified threshold θ, which was set to 0.5 in our implementation.

As described in the previous section, a patient may be diagnosed with more than one disorder. We approached this multi-labeling classification problem using the following two methods:

- Problem transformation: A binary relevance transformation method (7) was applied to transform the original problem into five binary classification tasks and learn five corresponding classifiers, one for each disorder. The original dataset was therefore transformed into five datasets, each of which contained all notes of the original dataset and each note was labeled with a binary value corresponding to the diagnosis. We then trained the five models on the datasets by using the binary cross-entropy function to calculate the training loss and optimize the loss by the back propagation algorithm. The union of the outputs from the five classifiers was collected to determine whether the patient had any of the five disorders.
- Feature dependency: Although the above well-known transformation-based approaches were successfully employed in a variety of multi-labeling applications in the literature, they cannot model dependencies between labels. By changing the number of units in the output layer such that they equal the number of desired diagnoses, the same neural network architecture developed for the first method can learn the hidden dependencies among different diagnoses via its shared hidden layers to enable it to directly carry out multi-label tasks. This approach allows us to simply train one classifier that can output five diagnoses at the same time. During the training phase, the cross-entropy loss was used to calculate the loss over the five outcomes of the classifier instead of the binary cross entropy, and the loss was minimized by the backpropagation algorithm.

**Figure 3:**
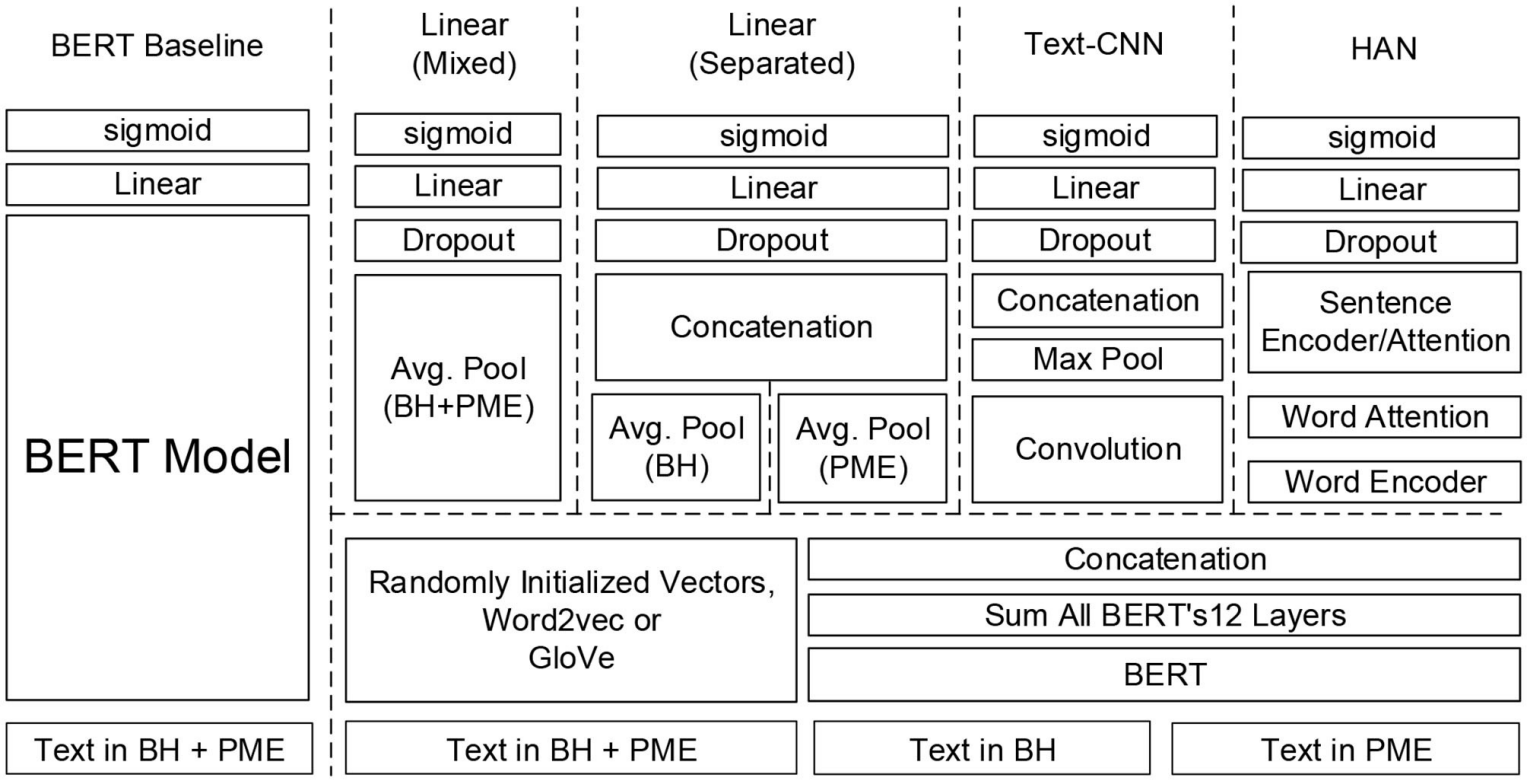
Deep learning text classification models developed in this study.

Based on the above two strategies, the five neural network architectures illustrated in Figure 3 were developed. The BERT model was implemented as a baseline because of its outstanding performance in several biomedical NLP tasks (3). In addition, we implemented three novel text classification architectures, including bag of words (BoW) linear models, textual convolutional neural network (CNN), and hierarchical attention network (HAN), with different pre-trained models for comparison because they are either the most common approaches used for clinical text classification or are becoming increasingly popular (8). As shown in Figure 3, two BoW linear models were implemented: one considers the texts from BH and PME individually to infer the diagnoses and the other considers the texts from both sections as a whole. The three architectures use the same layer structure to represent the sequence of input words by using either a simple look-up table over words or pre-trained language models, including word2vec, GloVe, and BERT, which are elaborated in the next section. For all of the developed models, the following sigmoid function was applied to compute the probability *p* for each disorder.

p(x|h)=sigmoid(Wh+b)

where ***W*** is the learned parameter matrix, which was fine-tuned with all the learnable parameters in the network jointly to maximize the log-probability of the correct disorders.

#### Baseline Model: BERT-Based Model Fine-Tuning

BERT (2) is a pre-trained deep learning model that learns bidirectional representations from large unlabeled text by jointly conditioning on both the left and right context with its transformer-based encoder architectures (9). As a result, the pre-trained BERT model can be fine-tuned with merely one additional output layer to create state-of-the-art models for a wide range of language understanding tasks. Therefore, we selected BERT as our baseline model and fine-tuned it for our dataset.

BERT was fine-tuned by using a sequence of 512 tokens as input, where the first and last tokens are always special tokens [(CLS) and (SEP)]. To use each summary for training and prediction, the tokens were collected from BH first followed by PME. For inputs consisting of fewer than 512 tokens, the special token (PAD) was used to pad each sequence to the same length; otherwise, the tokens that exceeded the length were truncated to meet the requirement of BERT. Inspired by Adhikari et al. (10), during the fine-tuning training process, the final hidden state *h* from the encoder corresponding to the special token (CLS) is considered as being representative of the entire sequence. The representation then passes through a linear layer with the sigmoid function to form a classifier that can generate the probability for each disorder.

#### Bag of n-Grams Linear Network

Representing text as BoW and using it as features for training linear classifiers, such as support vector machines (SVMs) (11) is a simple and efficient method to solve text classification problems. Inspired by Grave et al. (12), we implemented two linear models based on deep learning and exploited the *n*-gram features to capture information about the word order. The architectures of the two models were similar. The first layer is a sequence representation layer with dimension *d* to represent the given sequence of tokens. The representations are then averaged to form a fixed length vector with a length equal to *d*, which is considered to be able to capture the information of the given sequence. The vector is regularized by a dropout layer and, in turn, feeds to a linear layer we developed for the last layer of the BERT baseline model. The difference between the two developed models is that one model considers the texts in the BH and PME sections separately and uses two different sequence representation layers to encode the information, whereas the other uses one sequence representation layer to encode texts from both sections. We developed the variations because we consider it necessary to assign different weights to the information from the two sections. Our experimental results revealed that the PME section may include noisy information such that the model with separated input performed more accurately.

#### Textual Convolutional Neural Network

CNNs have been successfully and widely used in image classification tasks. One of the first attempts to apply CNNs to classify textual data was proposed by Kim (13) and recently by Yang et al. (14) for the automatic diagnosis of six diseases. Dai and Jonnagaddala (15) used a CNN to determine positive valence symptom severity in psychiatric evaluation records. We implemented the text-CNN model by applying convolutions to the represented sequences from both sections. Each convolution can be considered as a feature extractor that uses a kernel to capture implicit linguistic properties buried in the represented text within a context window of length *l*. In our implementation, context windows with lengths of 2, 3, 4, and 5 were included to capture the bigram, trigram, four-gram, and five-gram, respectively. The max-pooling layer that follows the convolutional layer subsamples the extracted features to obtain the maximum activation values. Through backpropagation, the combination of the above two layers enables us to capture the most prominent features for determining the diagnoses. Similar to the BoW linear models, all outputs from the max-pooling layer are concatenated to form a fixed-length feature vector, which is then fully connected to a linear layer with dropout to generate the outcome.

#### Hierarchical Attention Network

The HAN was designed by Yang et al. (16) specifically for document classification. HAN employs two hierarchical attentions, one for the word level and another for the sentence level, to capture the hierarchical structures of a document. We followed their design to implement the network. We used two encoders based on a bidirectional gated recurrent unit (GRU) to summarize (1) the information of the entire sequence of the represented words and (2) the entire sequence of sentences represented by the attended scores generated by the former encoder. Our model differs from that of Yang et al. in that the aforementioned sigmoid function was used in the last layer of our model because our task is a multi-labeling problem.

### Pre-trained Techniques

The input layers of all of the above models represent the given sequence of words in terms of vectors. Word embedding is a well-known NLP method, which maps each word into a dense vector of floating point values instead of one-hot encodings, to reduce the dimensionality of the input features and capture the relationships among each word at the same time. Pre-trained word embeddings are currently an essential component in state-of-the-art NLP systems. In this study, we considered two popular word embedding methods, word2vec, proposed by Mikolov et al. (17) and GloVe (18). In practice, both embeddings have demonstrated their ability to capture linguistic regularities in several NLP tasks; the former is more effective on certain datasets (19) and the latter on others (20). Word2vec has two model architectures to produce word embeddings in an unsupervised learning manner: continuous bag-of-words and continuous skip-gram. Herein, we used skip-gram to generate a pre-trained word2vec model from our training set because its architecture considers the words surrounding a target word as an entire context for prediction, which makes it more suitable for learning embeddings from a small dataset.

A potential drawback of the embeddings generated by the above approaches is that the contextual meaning of a word cannot be encoded because each word is represented by a vector with a fixed numerical value vector. This deficiency prompted researchers to propose a variety of pre-trained models that can be trained in an unsupervised manner and are simultaneously able to capture the contextual meaning of the words presented in texts. Early attempts include ULMFiT (Universal Language Model Fine-Tuning) (21) and ELMo (Embeddings from Language Models) (22), although the most successful model would have to be BERT (2). At least two ways exist in which to adapt the pre-trained BERT model to a specific task. The first is the way we presented in section Baseline Model: BERT-based Model Fine-Tuning to create the baseline model by directly fine-tuning BERT on our dataset. Another is to use BERT as a feature extractor to generate contextual word embeddings of which the values vary depending on their context. The parameters of the BERT model were frozen during training. The rationale is that the BERT model uses several layers of transformer encoders, and each output per token from each layer can be considered as a representation for that token. Devlin et al. (2) identified several ways to combine the outputs of each layer to form contextualized embeddings. As shown in Figure 3, we chose to sum the outputs of all the BERT layers to generate a fixed length embedding for each word in a sentence. The BERT embeddings were generated sentence-by-sentence and concatenated such that they are analogous to the results of traditional word embedding methods.

### Imbalance Issues

As shown in Figure 1, the annotations for four of the five disorders in our corpus are imbalanced, which could pose a significant obstacle in the way of the development of reliable classifiers. To address this issue, we applied a cost-sensitive learning approach (23, 24) by adding more costs when misclassifying minority cases. The most popular heuristic approach to estimate the cost directly from the training set is the use of the imbalance ratio, which is defined as the number of majority class examples divided by the number of minority class examples (25). For example, the imbalance ratio for “Schizophrenia” estimated on the training set is 7, thus the cost for incorrectly classifying a patient as not having “Schizophrenia” was set to 7.

### Evaluation Metrics

The micro- and macro- precision (P), recall (R), and F_1_-measures (F) were defined as follows and were used to evaluate the performance of the developed models. Precision and recall are also known as positive predictive value (PPV) and sensitivity (SEN), respectively.

$$\begin{array}{l} \text{Micro−Precision/PPV}=\frac{\sum_{l=1}^{5}\text{T}{\text{P}}_{l}}{\sum_{l=1}^{5}({\text{TP}}_{l}+{\text{FP}}_{l})},\\ \text{Micro−Recall/SEN}=\frac{\sum_{l=1}^{5}\text{T}{\text{P}}_{l}}{\sum_{l=l}^{5}({\text{TP}}_{l}+{\text{FN}}_{l})},\\ \text{Micro−F−measure}=2\frac{\text{Micro−PPV }\times \text{ Micro−SEN}}{\text{Micro−PPV }+\text{ Micro−SEN}},\\ \text{Macr−Precision/PPV}=average(\text{Per−class PPV}),\\ \text{Macro−Recall/SEN}=average(\text{Per−class SEN}),\\ \text{Macro− F− measure}=average(\text{Per−class F−measure}) \end{array}$$

In the above formulae, TP_*l*_, FP_*l*_, and FN_*l*_ represent the number of true positives (TPs), false positives (FPs), and false negatives (FNs) for diagnosis *l*, respectively. The per-class PR-scores can be calculated by using the same micro-PR formulae, but considering only one diagnosis at a time.

In general, we prefer models with higher precision and recall scores. However, a trade-off usually exists between precision and recall when machine-learning models are developed. The F-score provides a single metric to summarize the performance of a model in terms of PR scores by the harmonic mean. The F-score and PR metrics are widely used in NLP to evaluate the performance of text classification systems. We reported two types of F-measure obtained by using micro- and macro-averaging, respectively. We used the arithmetic mean of class-wise F-scores to calculate macro-averaging because it is significantly more robust on an imbalanced dataset (26). We preferred models with higher macro-averaging to those with higher micro-averaging because the latter are biased by class frequency.

## Results

### Experiment Design and Configurations

We designed two experiments with a variety of configurations to study the effectiveness of fine-tuning BERT on our dataset and to compare their performance with that of the three text classification networks along with different pre-trained models. All of the networks were implemented using PyTorch running on RTX 2080Ti GPUs. We used 20% of the original training set as the validation set and 80% as the training set. The validation set, which was not used for training, was used to determine the optimal parameters without overfitting the training set. Mini-batch gradient descent along with the Adam optimizer (with β_1_ = 0.9 and β_2_ = 0.999) was used to learn the parameters. The number of epochs was set to 500, and an early termination strategy was used if the F-scores were no longer observed to improve or the loss became zero on the validation set. We set a patience value of 30 to wait before applying early termination. For consistency, if not mentioned, we used the same set of hyper-parameters and a fixed random seed across all experiments.

### Results of Fine-Tuning BERT

Here, we examined the performance of fine-tuning pre-trained language models based on BERT on our dataset. The following four BERT-based models were considered: (1) the BERT_BASE_ uncased model,^2^ which contains 12 transformer encoder stacks, 12 self-attention heads with a hidden size of 768. (2) The ROBERTa_BASE_ (27) model,^3^ which was based on BERT_BASE_, but trained with enhanced training strategies over more data. (3) The DistilBERT_BASE_ uncased model,^4^ a light version of the original BERT, which was trained by using knowledge distillation (28). (4) The ALBERT_BASE_ model,^5^ which is another lite version of BERT trained by using parameter-reduction techniques (29). We empirically set the maximum number of epochs to 10 and saved the best model on the validation set for testing. During training, the batch size was set accordingly to ensure that the GPU memories were fully utilized. Tables 1, 2 provide the test set results of the models trained with different multi-labeling strategies.

**Table 1:** Performance of the BERT-based models with problem transformation on the test set.

**Diagnosis**	**Metric**	**Configuration**							
		**BERT**	**Distill**	**ALBERT**	**ROBERTa**	BERT*	Distill*	ALBERT*	ROBERTa*
Major depressive disorder	P	0.622	0.684	0.600	**0.803**	0.690	0.780	0.574	**0.803**
	R	0.578	0.684	**0.894**	0.859	0.660	0.684	0.473	0.859
	F	0.600	0.684	0.718	**0.830**	0.680	0.728	0.519	**0.830**
Schizophrenia	P	0	0.571	0	0	**0.600**	0.250	0.333	0
	R	0	**0.333**	0	0	0.250	0.166	0.250	0
	F	0	**0.421**	0	0	0.352	0.200	0.285	0
Bipolar	P	0	**1.000**	0	0	0.333	0.666	0.666	0.428
	R	0	0.100	0	0	0.100	0.200	**0.400**	0.300
	F	0	0.182	0	0	0.150	0.370	**0.500**	0.352
Minor depressive disorder	P	**0.461**	0.300	0	0	0	0.217	0.200	0
	R	**0.352**	0.176	0	0	0	0.294	0.058	0
	F	**0.400**	0.222	0	0	0	0.250	0.090	0
Dementia	P	0.777	**1.000**	0	0	0.666	0.727	0.250	0
	R	0.583	0.333	0	0	0.166	**0.666**	0.083	0
	F	0.666	0.500	0	0	0.266	**0.695**	0.125	0
Micro-Avg.	F	0.494	0.545	0.530	0.488	0.5	0.551	0.400	**0.584**
Macro-Avg.	F	0.334	0.446	0.146	0.167	0.31	**0.456**	0.311	0.238

*The asterisk indicates model configurations that include the use of weighted cost. Values in boldface indicate the highest PRF-scores*.

**Table 2:** The performance of the BERT-based models with feature dependency on the test set.

**Diagnosis**	**Metric**	**Configuration**							
		**BERT**	**Distill**	**ALBERT**	**ROBERTa**	BERT*	Distill*	ALBERT*	ROBERTa*
Major depressive disorder	P	0.632	0.600	0.636	0.682	0.571	0.611	0.691	**0.772**
	R	0.632	0.632	0.860	0.789	**0.982**	0.965	0.825	0.772
	F	0.632	0.615	0.731	0.748	0.723	0.748	0.752	**0.772**
Schizophrenia	P/R/F	0	0	0	0.000	0	0	0	0
Bipolar	P/R/F	0	0	0	0.000	0	0	0	0
Minor depressive disorder	P	0	0	0	**1.000**	0	0	0	0.667
	R	0	0	0	**0.118**	0	0	0	**0.118**
	F	0	0	0	**0.211**	0	0	0	**0.200**
Dementia	P	0	0	0	0	0	0	0	**1.000**
	R	0	0	0	0	0	0	0	**0.250**
	F	0	0	0	0	0	0	0	**0.400**
Micro-Avg.	F	0.436	0.429	0.530	0.556	0.544	0.556	0.534	**0.573**
Macro-Avg.	F	0.126	0.123	0.146	0.150	0.145	0.150	0.150	**0.274**

*The asterisk indicates model configurations that include the use of weighted cost. Values in boldface indicate the highest PRF-scores*.

Overall, we can observe that the use of cost-sensitive learning improves the generalization of the models on different diagnoses, which yields improved macro-avg. F-scores. ROBERTa obtained the best micro-avg. F-scores in both multi-labeling strategies because it performed more accurately in cases of major depressive disorder, which constitute most of the TP cases. The results also revealed that the problem transformation method is a more successful strategy when fine-tuning BERT-based models on our imbalanced multi-labeling dataset because most models trained with problem transformation produced higher macro-avg. F-scores than their counterparts using feature dependency. Most models trained with feature dependency cannot identify cases with dementia. Among all the studied models, DistilBERT with problem transformation had the best macro-avg. F-scores when cost-sensitive learning was applied.

### Results of Deep Learning Models With Different Pre-trained Methods

In this experiment, we investigated the effectiveness of combinations of the different pre-trained models and network architectures. The hyper-parameters set for the networks are listed in Table 3. For the pre-trained models, we used GloVe 6B-300d, which was trained on Wikipedia and Gigaword and empirically outperformed other word embedding methods on several medical NLP tasks (20, 30). The same BERT_BASE_ uncased model used for fine-tuning was chosen to generate the contextual embeddings. In addition, we included randomly initialized word embeddings as a baseline. The small dataset used in this study suggested using a smaller vector size to be learned for our embeddings. Thus, a vector size of 100 was chosen for the randomly initialized word embeddings and the pre-trained model generated by skip-gram. In addition, for the linear models with BERT embedding, we excluded the *n*-gram features because we considered the BERT embedding itself capable of providing sufficient information for learning. Finally, we noticed that the HAN-BERT model could not be fitted acceptably to the validation set; therefore, the dropout rate was specifically set to 0.

**Table 3:** Hyper-parameters used for the three models.

**Network architecture**	**Hyper-parameters**	**Shared hyper-parameters**
Linear (mixed/separated)	Max number of sequences: 2,048	Dropout rate: 0.5 Max number of vocabulary: 25,000 Minimum word frequency: 5
CNN	Max number of sequences: 2048 Number of kernels: 100 Kernel size: (2, 3, 4, 5)	
HAN	Max number of sentences: 100 Max number of words per sentence: 1,000 (BERT: 512) Encoder GRU hidden size: 250 Encoder GRU hidden layers: 2 Attention hidden size: 250	

**Figure 4:**
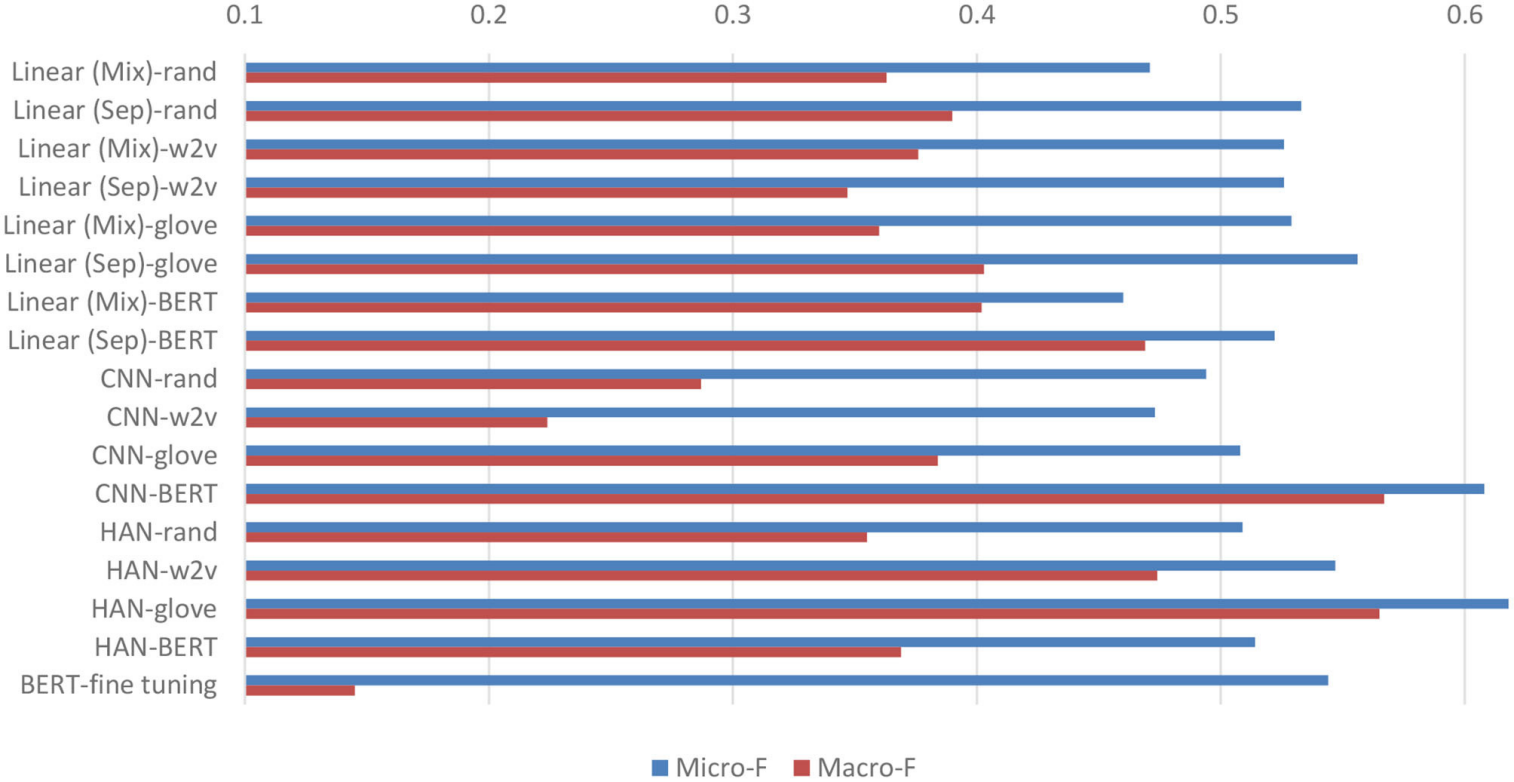
Micro- and macro-average F-scores on the test set of all developed models. “Linear (Mix)” signifies the linear model that considers the BH and PME sections in combination, whereas the “Linear (Sep)” model considers them separately. The figure also includes the performance of BERT* listed in Table 2 as a baseline (BERT-fine tuning) for comparison.

Figure 4 compares the micro- and macro-average F-scores of all developed models on the test set based on the feature dependency strategy. Overall, the models with the pre-trained techniques yield higher F-scores than those with random initialization. Compared with the fine-tuned BERT baseline, all models have higher macro-avg. F-scores. However, the micro-avg. F-scores of the models with random initializations are lower than that of the fine-tuned BERT. The results indicated that the pre-trained techniques enable the developed networks to learn more effectively than fine-tuned BERT from our small and highly imbalanced multi-labeling dataset.

In the case of the linear networks, the models with the proposed separation of the two sections demonstrated superior performance. In particular, the models with GloVe had higher micro-avg. F-scores because their performance in terms of classifying major depression cases was more accurate. On the other hand, models based on BERT had higher recalls in classifying other disease cases, resulting in higher macro-avg. F-scores. Among the text-CNN models, the model with BERT exhibited the highest micro-/macro-avg. F-score. Compared with the performance of all other models, it also demonstrated the highest F-scores of 0.667 and 0.431 for classifying schizophrenia and minor depression cases, respectively. Finally, for the HAN models, the best-performing network was based on GloVe, which had the highest micro-avg. F-score of 0.618 among all the developed models.

We noticed that the results of the models trained with the problem transformation strategy exhibited a very similar phenomenon to that observed in the models trained with feature dependency. In general, the models with GloVe or BERT outperformed those with other embeddings under the same network architecture. The best micro-avg. F-score was achieved by the linear (Sep) model with micro- and macro-avg. F-scores of 0.584 and 0.481, respectively. The HAN-glove model with a micro-avg. F-score of 0.542 had the highest macro-avg. F-score of 0.510.

**Figure 5:**
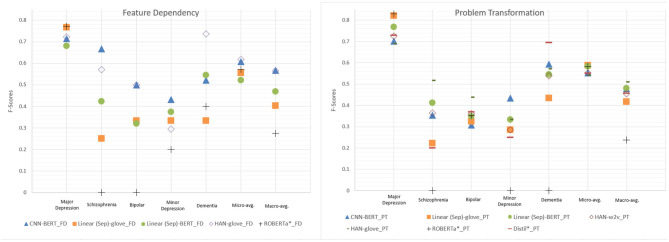
Performance comparison of the top performing models selected from the developed network architectures. PT and FD denote problem transformation and feature dependency, respectively. The fine tuning approaches (ROBERTa*_PT/FD and Distill*_PT) with the best micro- and macro-avg. F-scores are also included for comparison.

Figure 5 compares the performance of the top performing models trained with problem transformation or feature dependency. Overall, we observed that, based on the same network architecture, the models trained by feature dependency tended to deliver more optimal performance than those trained by problem formulation. Both the highest macro- and micro-avg. F-scores were achieved by the HAN-glove model trained using feature dependency (HAN-glove_FD). Unlike fine-tuned BERT, which has limited capability to identify patients with disorders other than major depression, all models, except CNN-w2v and CNN-rand, were able to identify at least one case of each disease. The developed models can identify major depression, dementia and schizophrenia with acceptable F-scores (0.667~0.772). The F-scores for bipolar and minor depression were less satisfactory (0.5 and 0.545). The results are consistent with the corresponding Kappa values reported in our previous work for assessing the inter-annotator agreement in which we found that the agreement for bipolar disorder and minor depression was significantly lower than the others (4). The detailed PRF-scores for all developed models for the five diseases are available in Supplementary Tables 1, 2.

## Discussion

### Effectiveness of the Developed Models and Pre-trained Techniques

Transfer learning has been extensively used in computer vision with great success; however, it has not been widely used in NLP until recent years (21). In the field of NLP, transfer learning refers more specifically to the applications of pre-trained language representations that were created by general models trained on large corpora. In this study, we examined two main types of pre-trained techniques: fine-tuning and feature-based models, such as our linear models with BERT embedding. As in Figure 5, the fine-tuning approach ROBERTa^*^_PT achieved the highest PRF-scores of 0.803/0.859/0.830 when classifying major depression cases, thereby surpassing the best-performing feature-based model [Linear (Sep)-glove_PT] by 0.09 in terms of the F-score. However, it cannot identify minority cases such as schizophrenia, minor depression, and dementia. The best macro-avg. F-score was achieved by Distill^*^_PT, although it is lower than that of most of the top-performing feature-based models.

Peters et al. (31) demonstrated that the adoption of fine-tuning rather than feature-based approaches is more beneficial, yet our results suggest that building complex network architecture on top of the pre-trained model improved the performance on our dataset. However, we would like to caution the readers in two respects when interpreting the above observation. First, we do not argue that feature-based approaches are always superior to fine-tuning methods. This would depend on the characteristics of the dataset and the hyper-parameters used for the models. Compared with the relatively large datasets used in previous studies, our dataset is a multi-labeling corpus and is small and highly imbalanced with respect to four out of the five disease labels. Second, we employed a cost-sensitive learning approach with the same weights across all models to address the imbalance issue because this approach has been demonstrated to be effective for addressing the imbalance problem in the medical domain (32) and several multi-class datasets with varying levels of imbalance (33). However, the best weights may vary depending on the network architectures and the employed pre-trained techniques. Hence, improved results may be obtained experimentally by using a hyper-parameter search (34). Furthermore, in addition to cost-sensitive learning, a variety of methods, such as oversampling and data augmentation, are available to address imbalance problems (23, 35). Different imbalance strategies may lead to diverse conclusions. We aim to address this issue in future work.

**Figure 6:**
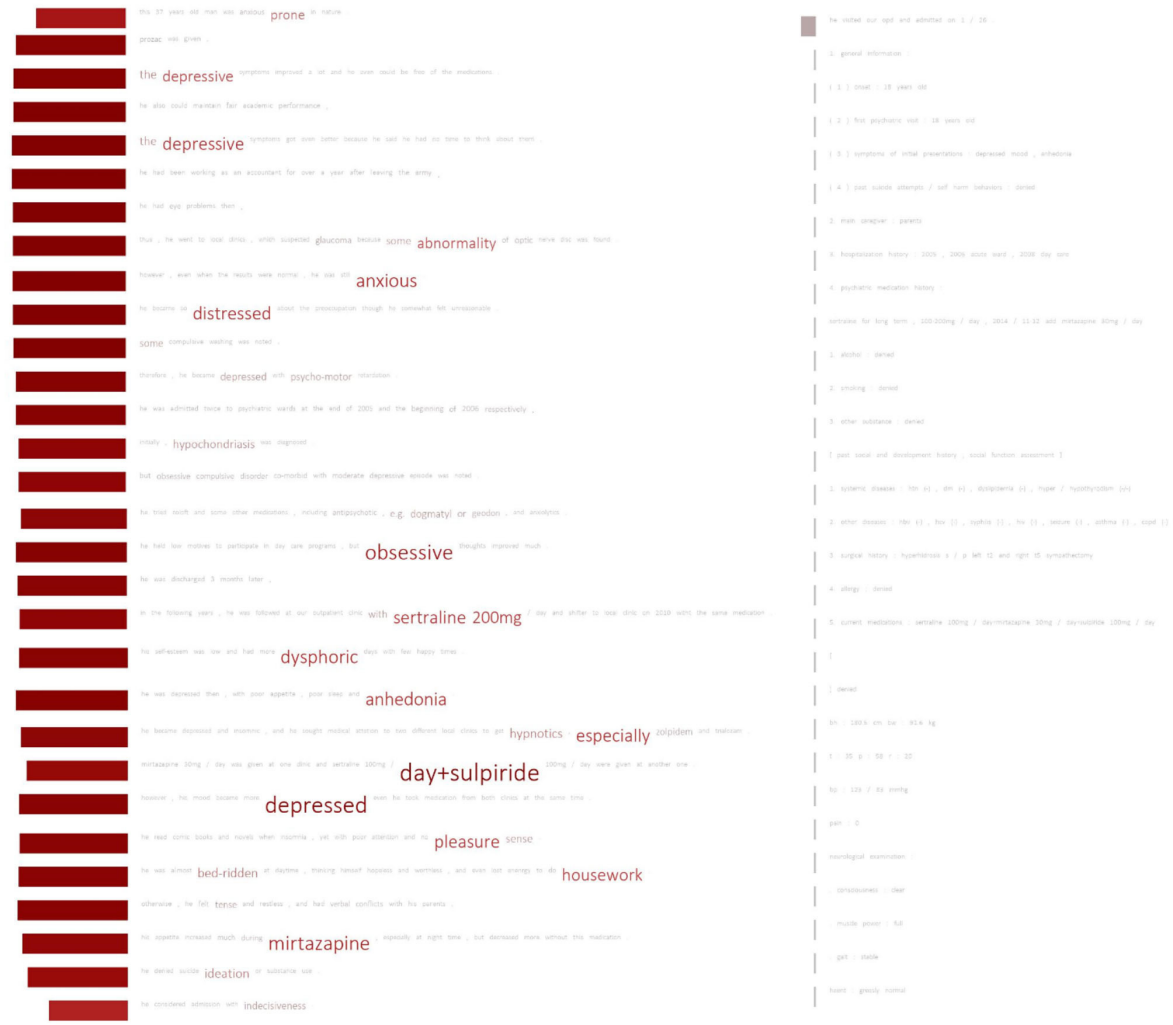
Important terms used by the HAN model for classifying a patient with major depressive disorder. The font size of a word indicates the attention score. Words with higher scores appear in larger font sizes. The rectangle shown in front of each sentence indicates the importance (attention score) of the sentence. Larger rectangles signify more important sentences.

Our results also responded positively to the main research question posed in the introduction section, namely that recent advanced pre-trained techniques could use small and imbalanced data to learn the diagnoses made by psychiatrists. As expected, the use of pre-trained techniques delivers more accurate results than random initialization. In particular, the micro-avg. and macro-avg. F-scores of the feature-based models can be improved by as much as 0.114 and 0.28, respectively, compared with those of the models with random initialization. Figure 6 graphically demonstrates an example of a major depression case correctly classified by our HAN model. Clearly, the model pays attention to important words associated with major depressive disorders, including “distress,” “anxious,” “obsessive,” “sertraline,” “dysphoric anhedonia,” “depressed,” and “pleasure.” In addition to terms related to depressive symptoms, terms that are not directly related but are related to medications, treatments, underlying personality traits, or comorbidities are identified, thus helping the model to correctly diagnose patients with major depressive disorder. The results indicate that when diagnosing major depression, the model not only depended on symptoms of the five traditional types of depression, but also on clues that could probably be related to depression that we never thought of before.

We also noticed that the model ignored most of the sentences from the PME section. We found that linear (Sep) models also have a similar tendency to assign larger weights to the words from the BH section. The PME section may include different semi-structural formats, such as lists or the results of biochemical examinations, which could be considered noise by those models. This can also explain why the linear (mixed) models performed worse.

**Figure 7:**
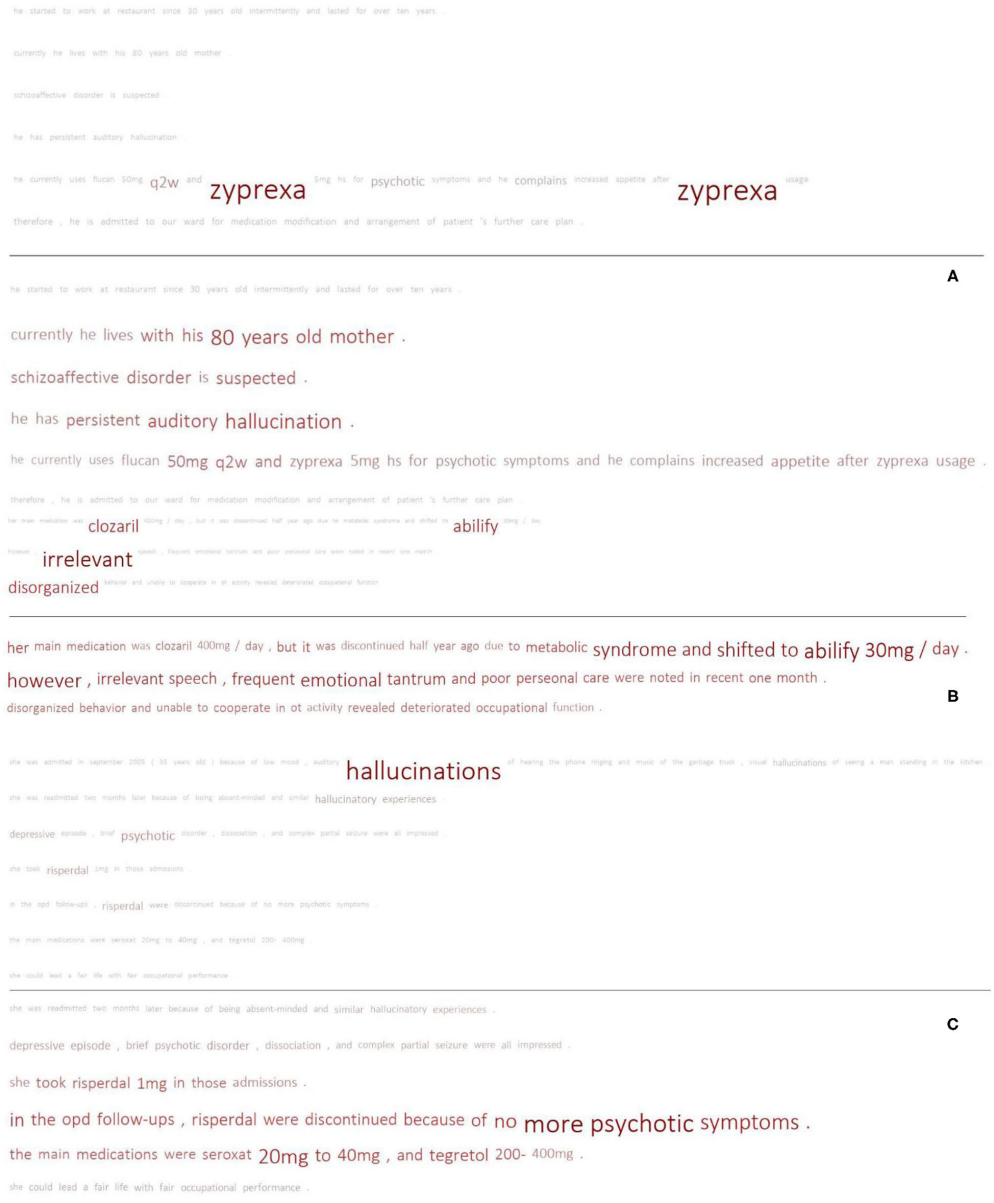
Comparison of the results of the pre-trained model and random initialization. Each sub-figure compares the results of HAN-glove_FD (upper) and HAN-rand_FD (lower). **(A)** TP vs. FN; **(B)** TP vs. TP; **(C)** FP vs. FP.

The macro-avg. F-scores of the randomly initialized models are apparently lower than those of the models with pre-trained techniques, particularly in the setting of feature dependency. This effectiveness seems to be the result of the knowledge provided by the pre-trained models, which cannot be inferred from the small training set at hand. This can be seen in Figure 7, which compares the schizophrenia results from two HAN models (rand and glove) trained with feature dependency. Figure 7A shows an example of an FN case of HAN-rand that could be correctly classified by HAN-glove. We can observe that the attentive words are vague for HAN-rand. The model focused on sentences containing key terms but could not concretize its attention on the terms. The glove model, in contrast, clearly focused on the key medication related to schizophrenia. The case shown in Figure 7B illustrates a similar phenomenon, but this time both models correctly classify it as a schizophrenia case.

**Figure 8:**
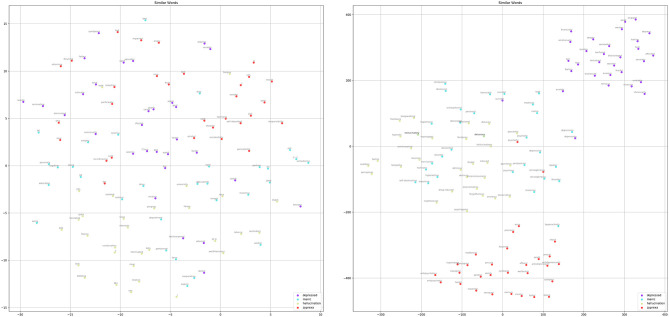
Comparison of the top-30 similar words for “depressed,” “manic,” “hallucination,” and “zyprexa” regarding the randomly initialized embedding of HAN-rand (left) and the pre-trained word embedding of the HAN-glove model (right).

We further applied the t-distributed stochastic neighbor embedding (t-SNE) algorithm (36) to visualize the similarity among the words based on the word embedding layers of the above two models after training on Figure 8. For the selected words, we extracted their top-30 similar words based on their embedding vectors and compressed them to a two-dimensional space but still retained similar words close together on the plane. The results clearly show that, after training, the randomly initialized word vectors did not show significant associations between similar words; however, the pre-trained embedding revealed meaningful associations. For example, “depressive” was linked to “suicide,” “anxiety,” “hurt,” etc.; on the other hand, “hallucination” was correlated with “auditory,” “telepathy,” “perceptual.” These words present the clinical symptom profiles, and a depressive mood might co-occur with the risk of suicide and anxious reactions. The experience of hallucination is generally associated with auditory sensation and this might also be interpreted by the patient as “telepathy.”

Because our dataset is small and the positive examples for disorders such as schizophrenia are limited, this may lead to bias in the optimization of the randomly initialized embedding. In contrast, the pre-trained models could provide implicit semantic knowledge to help the models make decisions. Figure 7C shows the FP results obtained by both models. Although both generated false alarms, the HAN-rand model ignored the negation marker (no more psychotic symptoms) and paid much more attention to the associated terms. On the other hand, the HAN-glove model ignores the negative statement, but considers several related indications, thereby resulting in an FP.

In summary, our results suggest that, for a multi-labeling dataset characterized by its small size and high imbalance, the feature-based model is superior to fine-tuning and pre-trained models are preferred. We also suggest using the feature dependency strategy to build multi-labeling models instead of using problem transformation because of its higher performance and simplicity in terms of the training process.

### Error Analysis

We discussed a few error cases regarding schizophrenia in the previous section. This section presents the results of our analysis of the remaining four disorders. First, we analyzed the errors for major and minor depressions. As stated in the previous section, deep learning models can be trained to make their decisions not only on the basis of symptoms of depression but also on other words that may provide clues. This suggests that it would be worthwhile comparing our method with the approach that identifies the major depressive cases by using the number of observed depressive symptoms only. Therefore, we used the depressive symptom recognizer developed in our previous studies (4, 37) to recognize the nine depressive symptoms including depressed mood, loss of interest, fatigue, appetite, sleep, psychomotor, poor concentration, worthless, and suicidality from the BH and PME section of an EHR. We then applied the rule to determine whether a patient has major depression if at least five unique symptoms are recognized in their EHR. Table 4 presents the results. The configuration “gold annotations” applied the same rule as the human annotators' depressive symptom annotations, which were annotated on the same dataset used in this study. Details of the symptom annotations can be found in our previous studies (4, 37).

**Table 4:** Test set results of the rule-based approach based on our previous studies to classify major depression cases.

**Configuration**	**Precision**	**Recall**	**F-score**
Recognizer	0.75	0.7368	0.7434
Gold annotations	0.7627	0.7895	0.7759

As indicated in Table 4, even with manual annotations, the PRF-scores only improve slightly. One of the reasons is that the annotation in our previous studies only focused on the BH section in patients' EHRs. Second, the clinical psychiatrists made their diagnoses by comprehensively and empirically reviewing the patient's treatment, chief complaint, and other detailed information included in the EHR, which cannot be captured by the employed rule. As shown in Figures 6, 7, the deep learning models attempt to comprehend the content based on their learned knowledge and exploit several factors to make their judgments which ultimately improve the recall or precision. For example, the best precision (0.892) and recall (0.947) among all developed models were achieved by CNN-BERT_PT and Linear (Mixed)-BERT_PT, respectively. We conducted an analysis based on the weights learned by the developed model assigned for each word, and found that the important terms in TP cases were mainly related to symptoms (e.g., bed-ridden, depressed, sad, or obsessively ruminated, worthlessness), medications (e.g., Cymbalta, Efexor, or antidepressant), and psychosocial stressors (e.g., separation or withdrawal). These findings support our claim that the classification made by the developed model is not only dependent on symptomatology, but also on the function and related treatment history.

However, the above feature could also increase the number of FPs. For example, “Lexapro” and “Zoloft” are commonly used to treat major depressive disorder, but they may also be used to treat generalized anxiety disorder. Certain terms used in EHRs may be related to several diagnoses. For example, “Efexor” could be linked to major depressive disorder, minor depression, and dementia. The associations among the five diseases and the learned important terms (e.g., constipation, mother-in-low, and remission) may be biased (or even unclear to us). Although these words might be related to underlying physical conditions, life situation, or treatment response, the small dataset at hand may not be able to allow the developed models to learn the implicit meaning among those terms and could lead to FPs or FNs.

## Data Availability Statement

The datasets analyzed in this article are not publicly available. Requests to access the datasets should be directed to Chi-Shin Wu, wuchishin@gmail.com.

## Ethics Statement

The studies involving human participants were reviewed and approved by National Taiwan University Hospital Research Ethics Committee (NTUH-201610072RINA). Written informed consent for participation was not required for this study in accordance with the national legislation and the institutional requirements.

## Author Contributions

H-JD and C-SW conceived the presented idea, supervised the findings of this work, and wrote the manuscript with support from C-HS. H-JD supervised the project. H-JD and Y-QL developed the network models and carried out the experiments. Y-CZ and C-KW pre-processed the dataset and developed rule-based methods for classifying major depression cases. C-SW acquired the dataset. C-JK, C-HS, and C-SW annotated the dataset. H-JD, C-SW, and C-HS discussed the results. All authors contributed to the article and approved the submitted version.

## Conflict of Interest

The authors declare that the research was conducted in the absence of any commercial or financial relationships that could be construed as a potential conflict of interest.

## Footnotes

^1^Our implementation is available at https://github.com/ken19980727/PsychiatricPatientScreening

^2^Available at https://github.com/google-research/bert

^3^Available at https://github.com/pytorch/fairseq/

^4^Available at https://github.com/huggingface/transformers/tree/master/examples/distillation

^5^Available at https://github.com/google-research/ALBERT
