# Supplementary material for: Deep Learning-Based Natural Language Processing for Screening Psychiatric Patients
Source: Front Psychiatry. 2021 Jan 15;11:533949. doi: 10.3389/fpsyt.2020.533949 (PMC7874001; doi:10.3389/fpsyt.2020.533949)
Supplement: Supplementary file 1 [file Data_Sheet_1.docx]

Detailed Precision Recall and F-scores for Developed Models

# Feature Dependency Formulation

**Supplementary Table 1.** The detailed precision, recall and F-scores of the developed networks based on feature dependency formulation.

|  | MDD | | | SCZ | | | BPD | | | mDD | | | DD | | | Micro | | | Macro | | |
| --- | --- | --- | --- | --- | --- | --- | --- | --- | --- | --- | --- | --- | --- | --- | --- | --- | --- | --- | --- | --- | --- |
|  | P | R | F | P | R | F | P | R | F | P | R | F | P | R | F | P | R | F | P | R | F |
| CNN-rand | 0.623 | 0.767 | 0.688 | 0.333 | 0.083 | 0.133 | 1 | 0.3 | 0.462 | 0 | 0 | 0 | 1 | 0.083 | 0.154 | 0.633 | 0.404 | 0.494 | 0.591 | 0.247 | 0.287 |
| CNN-w2v | 0.545 | 0.837 | 0.661 | 0 | 0 | 0 | 1 | 0.3 | 0.462 | 0 | 0 | 0 | 0 | 0 | 0 | 0.549 | 0.415 | 0.473 | 0.309 | 0.227 | 0.224 |
| CNN-glove | 0.589 | 0.767 | 0.667 | 0.429 | 0.25 | 0.316 | 0.429 | 0.3 | 0.353 | 0.385 | 0.294 | 0.333 | 0.5 | 0.167 | 0.25 | 0.529 | 0.489 | 0.508 | 0.466 | 0.356 | 0.384 |
| CNN-BERT | 0.58 | 0.93 | 0.714 | 0.778 | 0.583 | 0.667 | 0.5 | 0.5 | 0.5 | 0.324 | 0.647 | 0.431 | 0.545 | 0.5 | 0.522 | 0.519 | 0.734 | 0.608 | 0.545 | 0.632 | 0.567 |
| Linear-rand (Mixed) | 0.8 | 0.558 | 0.658 | 0.333 | 0.083 | 0.133 | 0.333 | 0.4 | 0.364 | 0.4 | 0.118 | 0.182 | 0.556 | 0.417 | 0.476 | 0.61 | 0.383 | 0.471 | 0.484 | 0.315 | 0.363 |
| Linear-rand (Sep) | 0.727 | 0.744 | 0.736 | 0.5 | 0.167 | 0.25 | 0.333 | 0.3 | 0.316 | 0.571 | 0.235 | 0.333 | 0.429 | 0.25 | 0.316 | 0.62 | 0.468 | 0.533 | 0.512 | 0.339 | 0.39 |
| Linear-w2v (Mixed) | 0.806 | 0.674 | 0.734 | 0.5 | 0.083 | 0.143 | 0.444 | 0.4 | 0.421 | 0.5 | 0.059 | 0.105 | 0.556 | 0.417 | 0.476 | 0.69 | 0.426 | 0.526 | 0.561 | 0.327 | 0.376 |
| Linear-w2v (Sep) | 0.727 | 0.744 | 0.736 | 0.5 | 0.083 | 0.143 | 0.6 | 0.3 | 0.4 | 0.5 | 0.118 | 0.19 | 0.667 | 0.167 | 0.267 | 0.69 | 0.426 | 0.526 | 0.599 | 0.282 | 0.347 |
| Linear-glove (Mixed) | 0.727 | 0.744 | 0.736 | 0.5 | 0.083 | 0.143 | 0.5 | 0.3 | 0.375 | 0.333 | 0.059 | 0.1 | 0.667 | 0.333 | 0.444 | 0.672 | 0.436 | 0.529 | 0.545 | 0.304 | 0.36 |
| Linear-glove (Sep) | 0.767 | 0.767 | 0.767 | 0.5 | 0.167 | 0.25 | 0.375 | 0.3 | 0.333 | 0.571 | 0.235 | 0.333 | 0.5 | 0.25 | 0.333 | 0.662 | 0.479 | 0.556 | 0.543 | 0.344 | 0.403 |
| Linear-BERT (Mixed) | 0.5 | 0.767 | 0.606 | 0.273 | 0.5 | 0.353 | 0.214 | 0.3 | 0.25 | 0.24 | 0.706 | 0.358 | 0.4 | 0.5 | 0.444 | 0.359 | 0.638 | 0.46 | 0.325 | 0.555 | 0.402 |
| Linear-BERT (Sep) | 0.646 | 0.721 | 0.681 | 0.333 | 0.583 | 0.424 | 0.267 | 0.4 | 0.32 | 0.29 | 0.529 | 0.375 | 0.429 | 0.75 | 0.545 | 0.441 | 0.638 | 0.522 | 0.393 | 0.597 | 0.469 |
| HAN-rand | 0.667 | 0.772 | 0.715 | 0.091 | 0.083 | 0.087 | 0.25 | 0.4 | 0.308 | 0.267 | 0.235 | 0.25 | 0.417 | 0.417 | 0.417 | 0.483 | 0.537 | 0.509 | 0.338 | 0.381 | 0.355 |
| HAN-w2v | 0.614 | 0.754 | 0.677 | 0.333 | 0.333 | 0.333 | 0.625 | 0.5 | 0.556 | 0.167 | 0.176 | 0.171 | 0.857 | 0.5 | 0.632 | 0.53 | 0.565 | 0.547 | 0.519 | 0.453 | 0.474 |
| HAN-glove | 0.694 | 0.754 | 0.723 | 0.5 | 0.667 | 0.571 | 0.5 | 0.5 | 0.5 | 0.294 | 0.294 | 0.294 | 1 | 0.583 | 0.737 | 0.607 | 0.63 | 0.618 | 0.598 | 0.56 | 0.565 |
| HAN-BERT | 0.609 | 0.737 | 0.667 | 0.4 | 0.333 | 0.364 | 0.25 | 0.3 | 0.273 | 0.286 | 0.118 | 0.167 | 0.75 | 0.25 | 0.375 | 0.529 | 0.5 | 0.514 | 0.459 | 0.348 | 0.369 |

# Problem Transformation Formulation

**Supplementary Table 2.** The detailed precision, recall and F-scores of the developed networks based on feature dependency formulation.

|  | MDD | | | SCZ | | | BPD | | | mDD | | | DD | | | Micro | | | Macro | | |
| --- | --- | --- | --- | --- | --- | --- | --- | --- | --- | --- | --- | --- | --- | --- | --- | --- | --- | --- | --- | --- | --- |
|  | P | R | F | P | R | F | P | R | F | P | R | F | P | R | F | P | R | F | P | R | F |
| CNN-rand | 0.891 | 0.719 | 0.796 | 0.75 | 0.25 | 0.375 | 0.455 | 0.5 | 0.476 | 0.17 | 0.882 | 0.286 | 0.208 | 0.833 | 0.333 | 0.3756345 | 0.6851852 | 0.4852459 | 0.4948 | 0.6368 | 0.4532 |
| CNN-w2v | 0.519 | 0.651 | 0.577 | 0 | 0 | 0 | 0.417 | 0.5 | 0.455 | 0.132 | 0.294 | 0.182 | 0.125 | 1 | 0.222 | 0.2731707 | 0.5185185 | 0.3578275 | 0.2386 | 0.489 | 0.2872 |
| CNN-glove | 0.769 | 0.702 | 0.734 | 0.5 | 0.333 | 0.4 | 0.25 | 0.3 | 0.273 | 0.281 | 0.529 | 0.367 | 0.571 | 0.333 | 0.421 | 0.5405405 | 0.5555556 | 0.5479452 | 0.4742 | 0.4394 | 0.439 |
| CNN-BERT | 0.892 | 0.579 | 0.702 | 0.6 | 0.25 | 0.353 | 0.25 | 0.4 | 0.308 | 0.345 | 0.588 | 0.435 | 0.533 | 0.667 | 0.593 | 0.5686275 | 0.537037 | 0.552381 | 0.524 | 0.4968 | 0.4782 |
| Linear-rand (Mixed) | 0.577 | 0.982 | 0.727 | 0.5 | 0.167 | 0.25 | 0.222 | 0.4 | 0.286 | 0.5 | 0.235 | 0.32 | 0.462 | 0.5 | 0.48 | 0.5142857 | 0.6666667 | 0.5806452 | 0.4522 | 0.4568 | 0.4126 |
| Linear-rand (Sep) | 0.804 | 0.789 | 0.796 | 0.333 | 0.167 | 0.222 | 0.179 | 0.5 | 0.263 | 0.163 | 0.882 | 0.275 | 0.556 | 0.417 | 0.476 | 0.3769634 | 0.6666667 | 0.4816054 | 0.407 | 0.551 | 0.4064 |
| Linear-w2v (Mixed) | 0.581 | 0.947 | 0.72 | 0.25 | 0.083 | 0.125 | 0.231 | 0.6 | 0.333 | 0.4 | 0.118 | 0.182 | 0.476 | 0.833 | 0.606 | 0.4899329 | 0.6759259 | 0.5680934 | 0.3876 | 0.5162 | 0.3932 |
| Linear-w2v (Sep) | 0.566 | 0.982 | 0.718 | 0.6 | 0.25 | 0.353 | 0.129 | 0.4 | 0.195 | 0.364 | 0.235 | 0.286 | 0.471 | 0.667 | 0.552 | 0.4601227 | 0.6944444 | 0.5535055 | 0.426 | 0.5068 | 0.4208 |
| Linear-glove (Mixed) | 0.804 | 0.789 | 0.796 | 0.5 | 0.083 | 0.143 | 0.263 | 0.5 | 0.345 | 0.4 | 0.118 | 0.182 | 0.417 | 0.417 | 0.417 | 0.6170213 | 0.537037 | 0.5742574 | 0.4768 | 0.3814 | 0.3766 |
| Linear-glove (Sep) | 0.836 | 0.807 | 0.821 | 0.333 | 0.167 | 0.222 | 0.222 | 0.6 | 0.324 | 0.75 | 0.176 | 0.286 | 0.455 | 0.417 | 0.435 | 0.6019417 | 0.5740741 | 0.5876777 | 0.5192 | 0.4334 | 0.4176 |
| Linear-BERT (Mixed) | 0.675 | 0.947 | 0.788 | 0.167 | 0.167 | 0.167 | 0.167 | 0.5 | 0.25 | 0.312 | 0.588 | 0.408 | 0.368 | 0.583 | 0.452 | 0.4508671 | 0.7222222 | 0.5551601 | 0.3378 | 0.557 | 0.413 |
| Linear-BERT (Sep) | 0.706 | 0.842 | 0.768 | 0.318 | 0.583 | 0.412 | 0.308 | 0.4 | 0.348 | 0.28 | 0.412 | 0.333 | 0.429 | 0.75 | 0.545 | 0.5033557 | 0.6944444 | 0.5836576 | 0.4166 | 0.5808 | 0.4812 |
| HAN-rand | 0.57 | 1 | 0.726 | 0.444 | 0.333 | 0.381 | 0.106 | 1 | 0.192 | 0.353 | 0.353 | 0.353 | 0.625 | 0.417 | 0.5 | 0.3596491 | 0.7592593 | 0.4880952 | 0.3804 | 0.6872 | 0.4304 |
| HAN-w2v | 0.57 | 1 | 0.726 | 0.286 | 0.583 | 0.364 | 0.267 | 0.4 | 0.36 | 0.205 | 0.471 | 0.286 | 0.5 | 0.583 | 0.538 | 0.4338624 | 0.7592593 | 0.5521886 | 0.349 | 0.5742 | 0.4548 |
| HAN-glove | 0.678 | 0.702 | 0.69 | 0.421 | 0.667 | 0.516 | 0.318 | 0.7 | 0.438 | 0.224 | 0.647 | 0.333 | 0.5 | 0.667 | 0.571 | 0.4484848 | 0.6851852 | 0.5421245 | 0.4192 | 0.6266 | 0.5096 |
| HAN-BERT | 0.714 | 0.702 | 0.708 | 0.417 | 0.417 | 0.417 | 0.189 | 1 | 0.317 | 0.239 | 0.647 | 0.349 | 0.6 | 0.25 | 0.353 | 0.4011628 | 0.6388889 | 0.4928571 | 0.3854 | 0.6698 | 0.4288 |
